# A High-Speed and Low-Offset Dynamic Latch Comparator

**DOI:** 10.1155/2014/258068

**Published:** 2014-07-09

**Authors:** Labonnah Farzana Rahman, Mamun Bin Ibne Reaz, Chia Chieu Yin, Mohammad Marufuzzaman, Mohammad Anisur Rahman

**Affiliations:** ^1^Department of Electrical, Electronic and Systems Engineering, Universiti Kebangsaan Malaysia, 43600 Bangi, Selangor, Malaysia; ^2^Mimos Berhad, 57000 Kuala Lumpur, Malaysia

## Abstract

Circuit intricacy, speed, low-offset voltage, and resolution are essential factors for high-speed applications like analog-to-digital converters (ADCs). The comparator circuit with preamplifier increases the power dissipation, as it requires higher amount of currents than the latch circuitry. In this research, a novel topology of dynamic latch comparator is illustrated, which is able to provide high speed, low offset, and high resolution. Moreover, the circuit is able to reduce the power dissipation as the topology is based on latch circuitry. The cross-coupled circuit mechanism with the regenerative latch is employed for enhancing the dynamic latch comparator performance. In addition, input-tracking phase is used to reduce the offset voltage. The Monte-Carlo simulation results for the designed comparator in 0.18 *μ*m CMOS process show that the equivalent input-referred offset voltage is 720 *μ*V with 3.44 mV standard deviation. The simulated result shows that the designed comparator has 8-bit resolution and dissipates 158.5 *μ*W of power under 1.8 V supply while operating with a clock frequency of 50 MHz. In addition, the proposed dynamic latch comparator has a layout size of 148.80 μm × 59.70 μm.

## 1. Introduction

Analog-to-digital converters (ADC) have become a significant element driving the semiconductor industry over the past few years. Increased integration of different functional blocks within a single chip makes ADCs more conventional and they are able to provide high speed with low power dissipation. In addition, some features of ADCs like small size processes, low power indulgences, and a reduced propagation delay make them more acceptable to the semiconductor industry. However, it is not straightforward to scale down transistor dimensions, as it requires high channel doping, gate-induced drain leakage, and band to band tunneling across the junction. The difficulty of short channel effects also needs to be controlled [1]. Moreover, analog circuit design happens to be more complex to carry out the necessity of reliability, where supply voltages need to be decreased according to the small dimensions of the transistors [2]. All these concerns apply to the most usable representative of the ADCs: the comparator.

The comparator is the key building block in the design process for ADCs. The comparators measure the smallest voltage differences in ADC's inputs, resolving the performance and the precision of any ADCs. An application that requires digital information recovery from analog signals, such as I/O receivers and radio frequency identification (RFID) memory circuits, widely uses high performance comparators to intensify a little input voltage to a big voltage level [3, 4]. Moreover, digital logic circuits can detect these signals within a short period. Therefore, a faster and precision-making comparator requires high gain and high bandwidth [5, 6].

Several structures of high-speed comparators exist, such as the multistage open loop comparator, the preamplifier latch comparator, and the regenerative latch comparator. Among the different structures, high resolution and high speed can be obtained easily by using the multistage open loop comparator. On the other hand, the latch-type comparator is the most usable one in the abovementioned applications due to its high-speed and low power consumption features. Latch-type comparators are able to accomplish decisions more rapidly with no static power indulgence and strong positive feedback [13]. Moreover, latch-type comparators are able to generate high gain in regeneration mode due to their positive feedback features. However, to design circuits for low voltage operations capable of decreasing the dynamic range of the inputs and the corresponding differential process [2, 14], the power dissipations in rail-to-rail operations are often increased. Consequently, the most vital limitations of the dynamic latch comparator are the kickback noises generated by high transmission currents [15]. In addition, employing a transmission gate can also induce spikes at the differential input voltage signals, which affects the performance of the dynamic latch comparator due to random noise, input offset voltages, and component mismatch.

In 2013, Zhu et al. designed an ultra-high-speed latched comparator with a controlled amount of positive feedback cell [16]. However, in this design, transmission gate switches are employed to reduce the power dissipation and the effect of charge injection. In other research work, Kapadia and Gandhi implemented a dynamic latch comparator using the CMOS charge-sharing concept, which also employed an extra buffer stage [17]. To obtain better performances, the uniqueness of the comparator, such as offset, input common mode range, propagation delay, and power dissipation, has been analyzed in both 130 nm and 90 nm technologies. Nevertheless, employing a buffer stage increases the power dissipation and the chip size. Singh and Gupta proposed a wideband flipped voltage follower (FVF) circuit using an inductive-peaking based bandwidth, which is able to engender low output impedance at high frequency [18]. On the other hand, this FVF design consumes more power. In 2013, Bhumireddy et al. introduced a novel latch-based comparator for successive approximation ADC with sub-32 nm double gate MOSFETs (DG-MOSFET) [19]. In this design, the regeneration time of the latch is enhanced by employing an extra positive feedback, which in turn increases the offset voltage and the propagation delay.

An amplifier is employed before the latched comparator, which decreases the offset voltages caused by the device mismatch. To achieve high gain to the output signal of the amplifier, a transmission gate can be utilized between the preamplifier and the latch, which in turn controls the signal path by using the insertion trend. Conventionally, a latch proceeded by preamplifier stages is utilized to employ a faster and accurate comparator [6, 20]. As a result, more area and power are dissipated by employing the preamplifier stages that also border the frequency bandwidth of the input signal. Miyahara et al. proposed a dynamic comparator with a self-calibration feature based on output averaging [21]. Moreover, in this design, a charge pump is required to regulate the corresponding input-referred offset voltage, making the approach inefficient. However, the involvement of this charge pump circuit limits its accuracy. In 2007, Verma and Chandrakasan proposed a novel structure for an offset compensated latch comparator. Nevertheless, convoluted timing necessities and a high number of offset annulment capacitors limit using this comparator in high-speed applications [22].

In this paper, a dynamic latch comparator is proposed based on differential pair input stages and one cross-coupled stage. Moreover, the proposed comparator is able to provide more high resolution and high speed with low power dissipation than conventional dynamic comparators at low supply voltage. The design is implemented in a Cadence Virtuoso 0.18 *μ*m CMOS process. The prelayout and postlayout simulation results prove that the circuit topology makes it applicable to work at very low supply voltage applications.

## 2. Design of Proposed Dynamic Latch Comparator

All the transistors should be properly matched in layout and biased in the saturation region to make a dynamic latch comparator more vigorous against mismatch and process variations. A fully differential dynamic latch comparator based on cross-coupled differential pairs is shown in Figure 6, which is based on the design of “Lewis-Gray” dynamic comparator [23].

**Figure 1:**
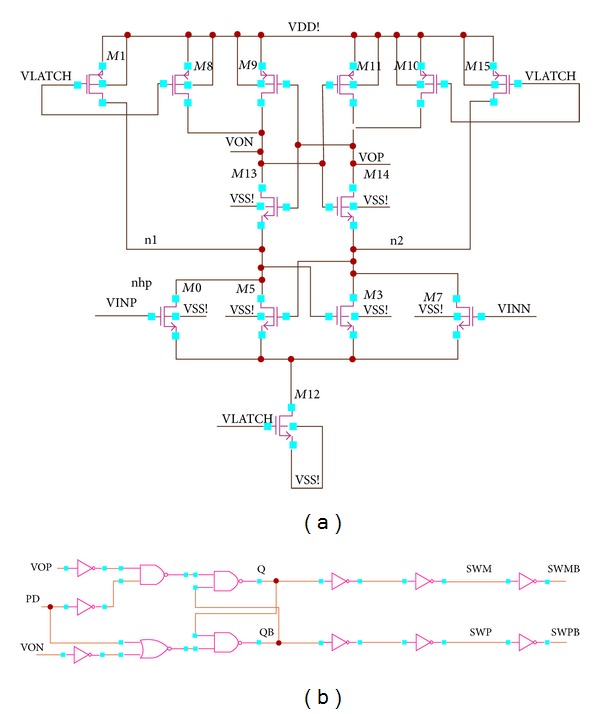
(a) Schematic diagram of the proposed differential pair dynamic latch comparator and (b) schematic diagram of the R-S flip-flop with digital signal PD.

In Figure 1, transistors* M*0,* M*3,* M*5, and* M*7 are utilized as the input circuitry. The overall latch circuitry consists of transistors* M*1,* M*8,* M*9,* M*10,* M*11,* M*12,* M*13,* M*14, and* M*15. In this proposed topology, the latch circuit is connected directly to the source coupled pairs* M*3 and* M*5 and the supply voltages* M*1,* M*8,* M*9,* M*10,* M*11, and* M*15, which makes the current sources switchable. To reset the S-R latch at the output of the comparator, a digital signal power down (PD) is used. When the comparator is off, VLATCH = low, the current source transistor* M*12 is switched off, and no current path exists from the supply voltage. The PMOS transistors* M*1,* M*8,* M*10, and* M*15 reset the outputs VON and VOP and nodes* n*1 and* n*2 to VDD. On the other hand, when VLATCH = high, the outputs are disconnected from the positive supply and switching current source* M*12 begins to conduct.* M*12 mainly determines the bias current of the input transistors* M*0,* M*7,* M*3, and* M*5. The cross-coupled NMOS pair* M*3 and* M*5 is utilized in the proposed topology to produce positive feedback that allows the output to switch faster. The switching mainly depends on the inputs VINP and VINN. In addition, the output signals SWM and SWP are unchanged (combine with the above). When the voltage VINP is bigger than VINM, the drain voltage of* M*0 will fall at a faster rate than the drain voltage of* M*7. Once the positive feedback from the cross-coupled NMOS transistors* M*5 and* M*3 kicks in, the node* n*1 will drop even faster and pull node VON low creating a logic low at the RS latch and the output* Q* = low. The overall circuit performance is depending on the devices size and dimensions which are shown in Table 1.

In this research, no offset cancelling techniques are introduced. However, there is a tradeoff between high speed and high accuracy (e.g., 8 bits) because of MOS device mismatches [24]. Effects of offset voltage can be reduced but cannot be circumvent completely. The total offset voltage of the comparator has the well-known dependency on the mismatch of the threshold voltage Δ*V*_*T*_, load resistance Δ*R*_*L*_, and transistor dimensions Δ*β* and the corresponding average values (*V*_*T*_, *R*_*L*_, and *β*):

(1) $$\begin{array}{c} V_{os}={\Delta V}_{T}+\frac{V_{gs}-V_{T}}{2}\left(\frac{{\Delta R}_{L}}{R_{L}}+\frac{\Delta \beta }{\beta }\right). \end{array}$$

In (1), the offset voltage is dominated by the Δ*β*, which is the mismatch of the transistor dimension, the overdrive voltage *V*_*gs*_ − *V*_*T*_. Moreover, the threshold voltage *V*_*T*_ also has an effect in (1). If the common mode voltage becomes lower (*V*_*gs*_, low), the offset is found to be smaller. The effect of the mismatches of the transistors from simulations* M*1,* M*8,* M*9,* M*10,* M*11,* M*13,* M*14, and* M*15 in this topology is not very critical. Moreover, the transistors in the input differential pair and the cross-coupled NMOS are significant, as these* M*0,* M*3,* M*5,* M*7, and* M*12 transistors determine the overdrive of the input differential pair, which is related proportionally to the *V*_*gs*_ − *V*_*T*_ in (1).

## 3. Results and Comparison

**Figure 2:**
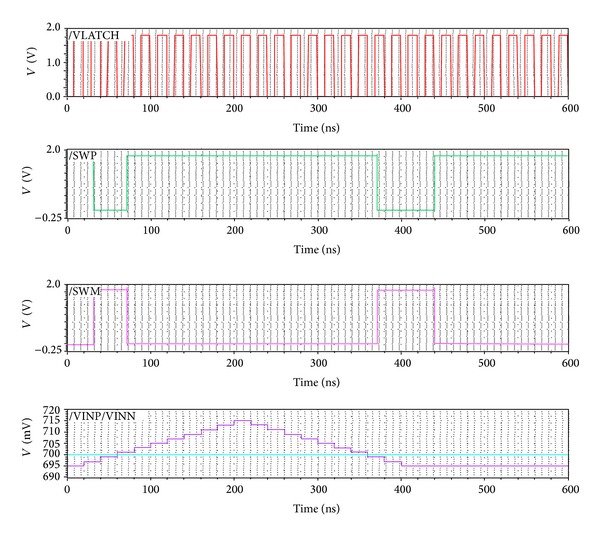
Transient simulation of the comparator input signals, VLATCH signal, and output signals (SWP and SWM) using Virtuoso Spectre.

The proposed dynamic latch comparator circuit has been verified using the SPECTRE simulator (CADENCE). The CADENCE Virtuoso in a 0.18 *μ*m CMOS process parameter is utilized in this design. The simulated behavior of the comparator is illustrated in Figure 2. It is observed from Figure 2 that with a 2 mV positive step size for the input VINP and keeping VINN fixed at 0.7 V, the proposed dynamic latch comparator can switch successfully. In this topology, the stepping of the input signal VINP (going up and coming back down) is used to check whether there is any hysteresis or not [25]. From the simulated results of Figure 2, it is found that when VINP > VINN (VINP = 695 mV and VINN = 700 mV) on the rising edge of the VLATCH signal, the proposed dynamic latch comparator can switch successfully. Similarly, switching also happens whenever VINP < VINN during VLATCH = high. In this case, the input signals VINP and VINN values are chosen in such a way to find out the output variation for small input changes.

**Figure 3:**
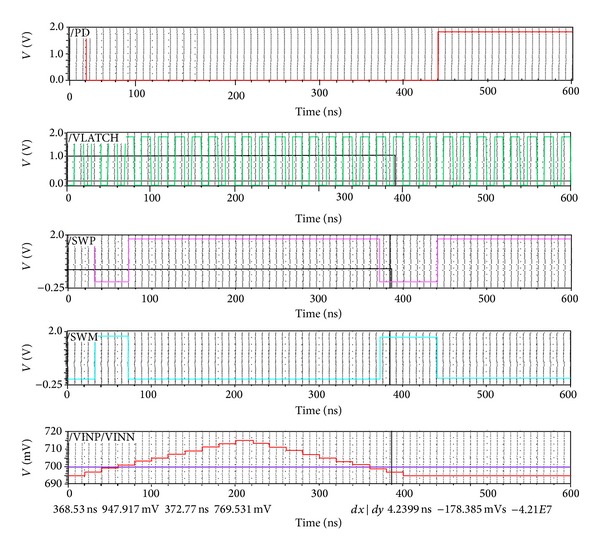
Propagation delay waveform between the VLATCH and SWP signal.

Figure 3 shows the propagation delay between the latch and the output signals. When input signal VINN > VINP and the VLATCH is high, output signal SWM is in the rising state and output signal SWP is in the falling state. The simulated result shows that during the rising edge of the SWP maximum propagation delay between the VLATCH and SWP signal is about 4.2 nS.

**Figure 4:**
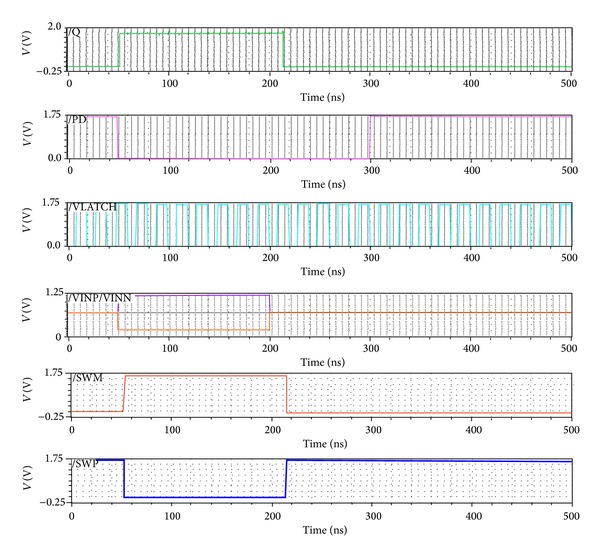
Corner analysis of the comparator input signals, VLATCH signal, and output signals (SWP and SWM).

To verify the robustness of the proposed dynamic latch comparator, different increments of environmental conditions like temperature, voltage clock frequency, and so forth need to be tested. To test the process variation, all 45 corners, 3 Vcc (1.7, 1.8, and 1.9), 3 temperatures (27, 0, and 90), and 5 corners (typical, snsp, snwp, wnsp, and wnwp) are analyzed for the proposed design. In addition, different stepping sizes of VINP and a fixed VINN at 0.7 V are analyzed.  Figure 4 shows that at different variations of Vcc and temperature the proposed dynamic latch comparator has been switching properly. Whenever VNP > VINN and VLATCH is high, SWM and SWP switch properly. Similarly, switching also happens whenever VINP < VINN during VLATCH = high.

**Figure 5:**
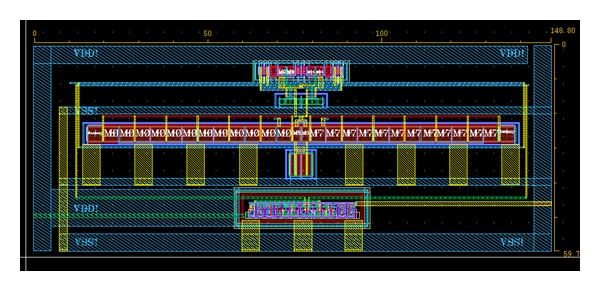
A layout design of the proposed dynamic latch comparator.

The chip layout is shown in Figure 5, where the chip occupies an area of 148.80 *μ*m × 59.70 *μ*m. In this layout, all the transistors are placed symmetrically to reduce mismatch in the parasitic capacitance.

**Figure 6:**
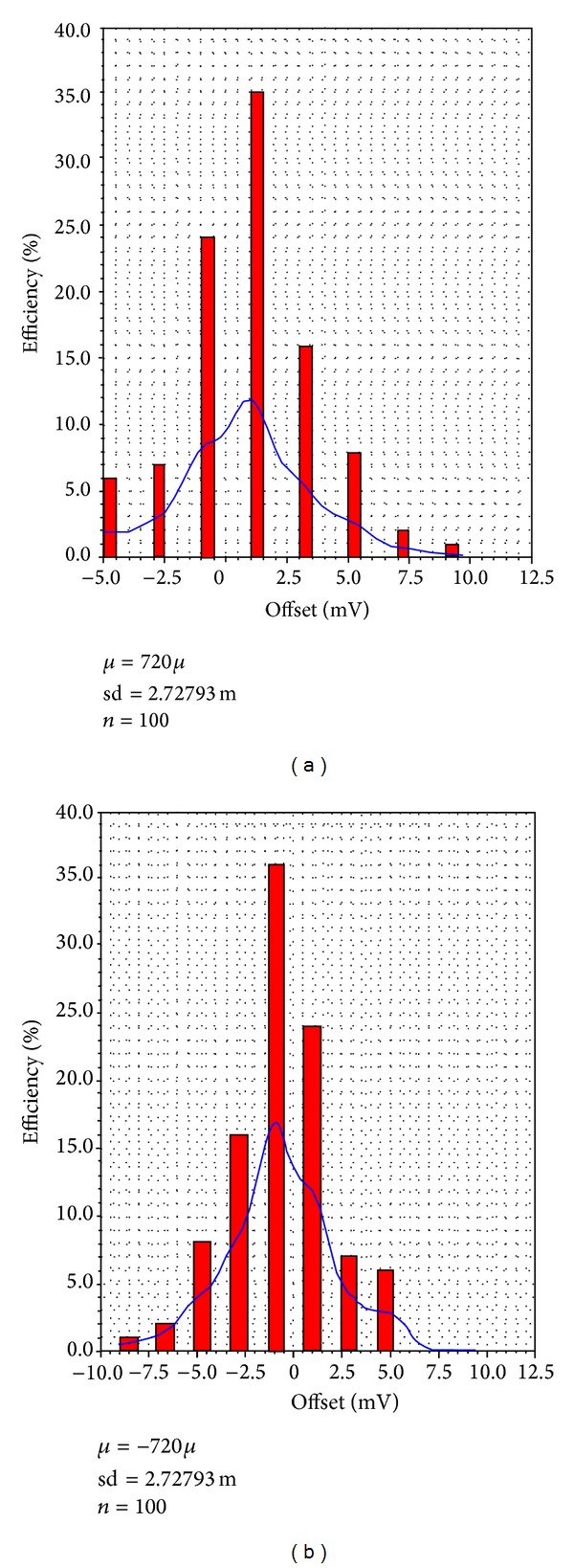
Postlayout Monte-Carlo simulation result with process and mismatch variation.

The postlayout Monte-Carlo simulation results for 100 runs are shown in Figure 6, which found that a higher offset value was obtained at a sampling frequency of 50 MHz using VDD 1.8 V with the overdrive voltage of 3.44 mV, which corresponds to 0.5 LSB at 8-bit precision.

Table 2 summarizes the proposed latch comparator performance with recently published research works. Compared to the research works of [7, 8], the proposed dynamic latch comparator has less offset voltage. The comparator of [7, 10] works only in 20 MHz sampling rate, whereas this design is able to run in 50 MHz sampling rate. Moreover, propagation delay is significantly lower than the research works published in [8, 10]. Apart from the research work of [12], this design has more resolution (8 bits instead of 7 bits).

To compare the performance of different comparators, a well-known figure of merit (FOM) is used [26]. Therefore, in this research, to measure the performance of the design, the FOM is calculated using the following equation:

(2) $$\begin{array}{c} \text{FOM}=\frac{P_{d}}{2^{n}*f_{s}}, \end{array}$$

where *P*_*d*_ is the power dissipation, *n* is the number of bits (resolution), and *f*_*s*_ is the sampling frequency of the comparator. From the comparison study of different recently published works as shown in Table 2, the proposed dynamic latch comparator has the lowest FOM energy dissipated per conversion among all the recently published research works.

In this research, the proposed dynamic latch comparator is able to work for 8-bit resolution, whereas the resolution of [10] is found to be 12 bits. To improve the overall resolution of the proposed dynamic latch comparator reduced offset voltage, different transistor sizing for reducing mismatch and layout methods in the realized chip can be implemented. In this research, *V*_*T*_ mismatches is reduced by using big transistors like* M*0,* M*3,* M*5, and* M*7. Mismatch among the devices can be found from the following equation:

(3) $$\begin{array}{c} \sigma_{\Delta VT}=\frac{A_{VT}}{\sqrt{WL}}. \end{array}$$

As a result, overdrive voltage has been increased and *V*_*gs*_ − *V*_*T*_ is decreased. Therefore, to improve the resolution of the proposed design, tail current of* M*12 can be reduced. However, the proposed design has removed the preamplifier stage and employed a dynamic latch, resulting in significant power saving, especially in flash and pipelined A/D architectures and RFID transponders.

## 4. Conclusion

A novel high-speed, low power, and low-offset dynamic latch-type comparator method is presented in this research work. The proposed design does not use any preamplifier stages before the latch stage, which reduces the power dissipation and the area dramatically. The corner analysis and the Monte-Carlo simulation results clearly reveal that the dynamic latch comparator is able to switch properly with different input stepping sizes. Though the proposed design has 8-bit resolution with 50 MHz sampling rate it consumes much lower power. Moreover, the comparison study shows that the novel design is able to operate at a higher clock frequency of 50 MHz with offset voltage 3.44 mV and propagation delay 4.2 nS in 1.8 V supply voltage, which is better than recently published research works.

## Figures and Tables

**Table 1:** Transistor dimensions used in this proposed topology.

Transistors	*W* (*μ*m)	*L* (*μ*m)	*m* factor
*M*0	4	4	12
*M*1	2	0.18	1
*M*3	4	2	1
*M*5	4	2	1
*M*7	4	4	12
*M*8	2	0.18	1
*M*9	4	2	1
*M*10	2	0.18	1
*M*11	4	2	1
*M*12	6	1	2
*M*13	2	1	1
*M*14	2	1	1
*M*15	2	0.18	1

**Table 2:** Comparison study of the proposed latch comparator performance.

References	[7]	[8]	[9]	[10]	[11]	[12]	This work
Year	2009	2010	2010	2011	2012	2013	
Technology (*μ*m-CMOS)	0.35	0.5	0.18	0.18	0.9	0.65	0.18
Supply voltage (V)	1.2	±1.5	1.8	1	1	1	1.8
Power *P*_*d*_ (*μ*W)	8.4	—	225	63.5	240	157	158.5
Sampling rate (MHz)	20	—	30	20	50	50	50
Resolution (bits)	8	—	8	12	6	7	8
Propagations delay (nS)	—	932^a^	—	26^a^	—	—	4.2
Offset voltage (mV)	3	24.2	—	0.0476	—	—	3.44
FOM (fj/conv)	1.64	—	29.2	0.77	150	28	0.7

^a^Measured value.
